# Expression pattern of NLRP3 and its related cytokines in the lung and brain of avian influenza virus H9N2 infected BALB/c mice

**DOI:** 10.1186/s12985-014-0229-5

**Published:** 2014-12-30

**Authors:** Meng Yu, Kaizhao Zhang, Wenbao Qi, Zhiqiang Huang, Jinhui Ye, Yongjiang Ma, Ming Liao, Zhangyong Ning

**Affiliations:** College of Veterinary Medicine, South China Agricultural University, Guangzhou, 510642 People’s Republic of China

**Keywords:** Avian influenza virus, H9N2, BALB/c mice, NLRP3, IL-1β, TNF-α

## Abstract

**Background:**

H9N2 avian influenza virus (AIV) becomes the focus for its ability of transmission to mammals and as a donor to provide internal genes to form the new epidemic lethal influenza viruses. Residue 627 in PB2 has been proven the virulence factor of H9N2 avian influenza virus in mice, but the detailed data for inflammation difference between H9N2 virus strains with site 627 mutation is still unclear. The inflammasome NLRP3 is recently reported as the cellular machinery responsible for activation of inflammatory processes and plays an important role during the development of inflammation caused by influenza virus infection.

**Methods:**

In this study, we investigated the expression pattern of NLRP3 and its related cytokines of IL-1β and TNF-α in BALB/c mice infected by H9N2 AIV strains with only a site 627 difference at both mRNA and protein levels at different time points.

**Results:**

The results showed that the expression level of NLRP3, IL-1β and TNF-α changed in the lung and brain of BALB/c mice after infection by V_K627_ and rV_K627E_. The immunohistological results showed that the positive cells of NLRP3, IL-1β and TNF-α altered the positive levels of original cells in tissues and infiltrated inflammatory cells which caused by H9N2 infection.

**Conclusions:**

Our results provided the basic data at differences in expression pattern of NLRP3 and its related cytokines in BALB/c mice infected by H9N2 influenza viruses with only a site 627 difference. This implied that NLRP3 inflammasome plays a role in host response to influenza virus infection and determines the outcome of clinical manifestation and pathological injury. This will explain the variable of pathological presentation in tissues and enhance research on inflammation process of the AIV H9N2 infection.

## Introduction

H9N2 avian influenza viruses (AIV) are widely circulating in both wild birds and domestic poultry populations since isolated from turkeys in 1966 and become a pandemic threat to human [1-4]. Evidences showed that this subtype of avian influenza virus had been transmitted from birds to mammals such as dogs, cats and pigs [5-7]. Birds and infected mammals are thought to be the reassortment for forming new strains based on this subtype AIVs [8,9]. H9N2 avian influenza virus caused low mortality and immunosuppression in chickens [10,11] and become a more prevalent pathogen in human populations since the first infection case of human in 1998 [12,13]. It is noteworthy that it can provide the inner genes to other AIV types and form new lethal influenza viruses to humans, such as H5N1, H7N9 and H10N8 [14-18] and co-circulates with the other AIV types during their prevalence [3,19]. The inflammation process of H9N2 infection may be slight respiratory symptoms in human [12,13], but the more serious clinical signs in mice [10,20-22]. Our previous reports showed that a single-amino-acid substitution in PB2 residue 627 changed the virulence of H9N2 avian influenza virus in mice and showed different pathological change in lung and brain [21,22]. The relationship of pathological changes associated with the NLRP3 and its related cytokines production need to be explored.

Nod-like receptors (NLRs), one of pathogen-associated molecular patterns (PAMPs), play a significant role in inflammation development in virus infection and regulate the host response to adaptive immunity [23]. Among different types of inflammasome, the NLRP3 inflammasome is well characterized in a variety of mammalian cells which is mainly composed of the NLRP3, the adaptor protein apoptosis-associated speck-like protein (ASC) and caspase-1. NLRP3 is the central component of the NLRP3 inflammasome which can recruit pro-caspase-1 with ASC [24]. With the controlling of cytosolic multiprotein complexes inflammasome protease caspase-1 has pivotal function in regulating the cleavage and maturation of pro-IL-1β, which is the inactive precursor of IL-1β, in response to a variety of agonists or stimuli [25]. IL-1β is an important cytokine with a broad range of biological activities. In influenza infection, IL-1β bind with its receptors leads to the activation of multiple cytokines, including TNF-α [26] which plays an important role in the early stages of host defense against influenza infection [27]. The expression pattern and histological distribution of NLRP3, IL-1β and TNF-α during the H9N2 avian influenza virus infection in mice are still unknown.

Here, we provided the basic data of expression pattern and histological distribution of NLRP3, IL-1β and TNF-α in lung and brain during infection in BALB/c mice by H9N2 avian influenza virus strains with only a difference at site 627 in PB2. This will allow us to further understand the function of NLRP3 inflammasome for the role of residue 627 in PB2 in mammalian hosts. To our knowledge, there are very few papers to show the expression profile and histological distribution change of NLRP3, IL-1β and TNF-α after H9N2 influenza virus infection.

## Results

### Virulence of avian influenza virus H9N2 in mice

The weight loss, clinical signs after inoculation with V_K627_ and rV_K627E_ avian influenza virus H9N2 in mice are consistent with our previous reports [21,22]. V_K627_ virus induced significant viral encephalitis while rV_K627E_ not and V_K627_ virus induced mild interstitial pneumonia while rV_K627E_ only induced very slight lung change [21,22].

### Expression changes of NLRP3, IL-1β and TNF-α during H9N2 AIV infection

**Figure 1:**
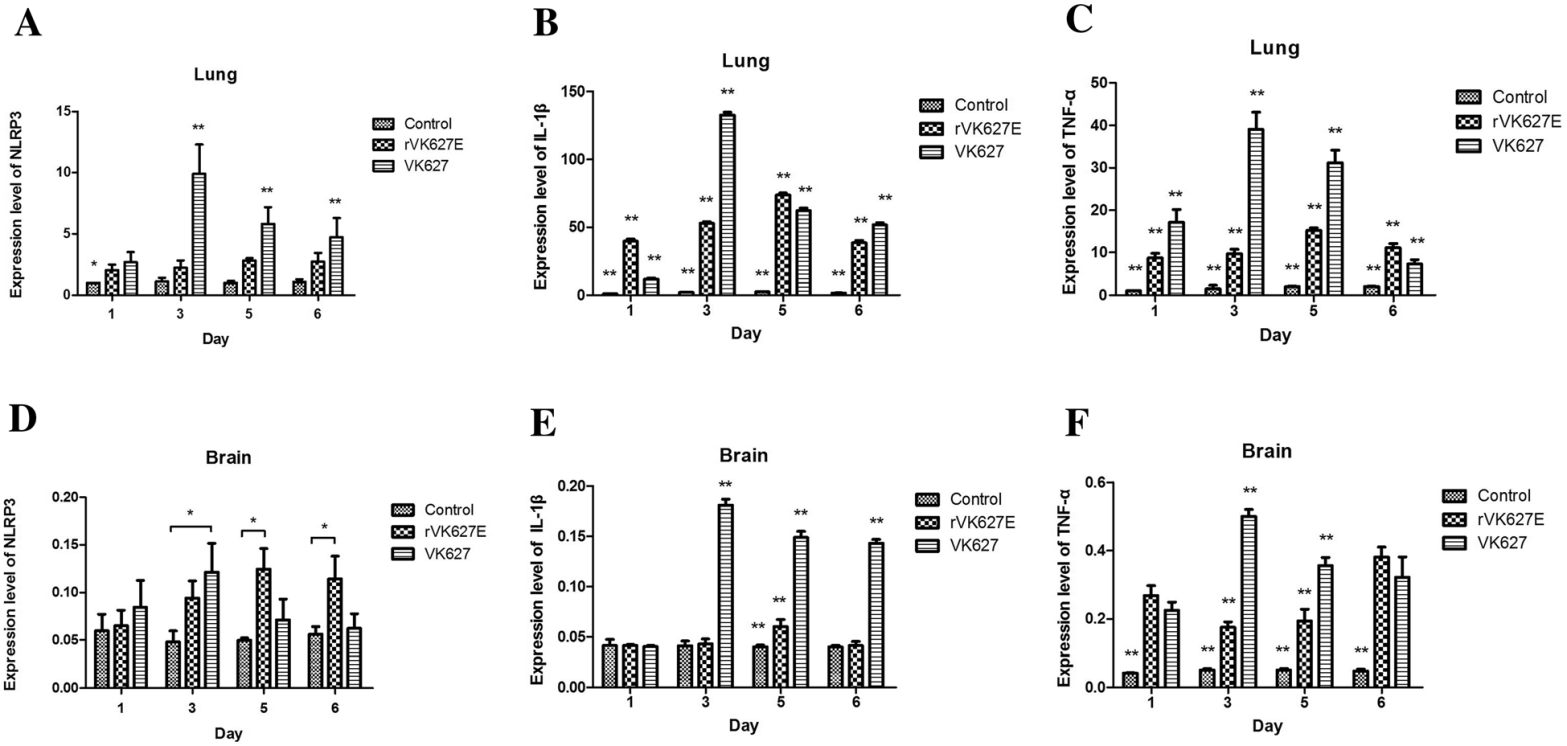
**Relative expression level of NLRP3, TNF-α and IL-1β in the lung and brain of control, rV**_**K627E**_ **and V**_**K627**_ **groups at different time points.** ^*^, P < 0.05; ^**^, P < 0.01. Data are represented as mean ± standard deviation (SD). All samples were tested in triplicate. **A**, **B** and **C**: Expression level of NLRP3, IL-1β and TNF-α in the lung of control, rV_K627E_ and V_K627_ groups at different time points, respectively; **D**, **E** and **F**: Expression level of NLRP3, IL-1β and TNF-α in the brain of control, rV_K627E_ and V_K627_ groups at different time points, respectively.

The expression level of NLRP3, IL-1β and TNF-α analyzed by quantitative real-time PCR altered in the lung and brain of the infected mice and differed with the control (Figure 1).

In the lung, expression level of NLRP3 increased after infection compared with the control. In V_K627_ group, the expression level of NLRP3 was sharply achieved its highest level at 3 dpi from the lowest at 1 dpi before continuously declined at 5 and 6 dpi. In rV_K627E_ group, the expression level of NLRP3 was gently achieved its highest level at 5 dpi from the lowest at 1 dpi. The expression level of NLRP3 in the control was significantly lower than rV_K627E_ and V_K627_ infection group (P < 0.05) at 1 dpi. The expression level of NLRP3 in V_K627_ group is very significantly higher than that of rV_K627E_ group at 3, 5 and 6 dpi (P < 0.01) (Figure 1A). Expression level of IL-1β in lungs of V_K627_ and rV_K627E_ group were very significantly higher than the control (P < 0.01). In V_K627E_ group, expression level of the IL-1β was lowest at 1 dpi and then sharply increased to its peak at 3 dpi before continuously declined at 5 dpi and 6 dpi. In rV_K627E_ infection group, the expression level of IL-1β rose from 1 dpi to 5 dpi which achieved its peak before declined to the lowest level at 6 dpi. The expression level of IL-1β in V_K627_ infection group was very significantly higher than rV_K627E_ group at 3, 5 and 6 dpi (P < 0.01) (Figure 1B). Expression level of TNF-α in the lung of V_K627_ and rV_K627E_ infection groups were very significantly higher than the control (P < 0.01). In V_K627E_ group, the expression level of TNF-α achieved its peak at 3 dpi and then continuously declined to its lowest at 6 dpi. In rV_K627E_ group, the expression level of TNF-α rose from the lowest level at 1 dpi to the highest level at 5 dpi before declined at 6 dpi. At all time-points, the expression level of TNF-α in V_K627_ infection group was very significantly higher than rV_K627E_ infection group(P < 0.01) (Figure 1C).

In the brain, expression level of NLRP3 rose after V_K627_ and rV_K627E_ infection compared with the control. The expression level of NLRP3 in V_K627_ infection group was higher than rV_K627E_ group and the control at 1 dpi and then achieved its peak at 3 dpi before declined at 5 and 6 dpi. In rV_K627E_ infection group, the expression level of NLRP3 continuously increased till achieving its highest level at 5 dpi. The expression level of NLRP3 in V_K627_ infection group at 3 dpi and in rV_K627E_ group at 5 dpi and 6 dpi were significantly higher than the control (P < 0.05) (Figure 1D). Expression tendency of IL-1β in brain was similar to NLRP3 and the expression level in V_K627_ group is very significantly higher than the other two groups at all time-points except 1 dpi. The expression level of IL-1β in rV_K627E_ group was very significantly higher than control group at 5 dpi (P < 0.05) (Figure 1E). Expression level of TNF-α in the control was very significantly lower than V_K627_ and rV_K627E_ infection group at all days detected (P < 0.01). Expression level of TNF-α in V_K627_ group were very significantly higher than rV_K627E_ group at 3 dpi and 5 dpi (P < 0.01). Different with NLRP3 and IL-1β, expression level of TNF-α in rV_K627E_ continually declined to its lowest level at 5 dpi from 1 dpi and then sharply rose to its highest level at 6 dpi. (Figure 1F).

### Immunohistochemical detection of NLRP3, IL-1β and TNF-α

**Figure 2:**
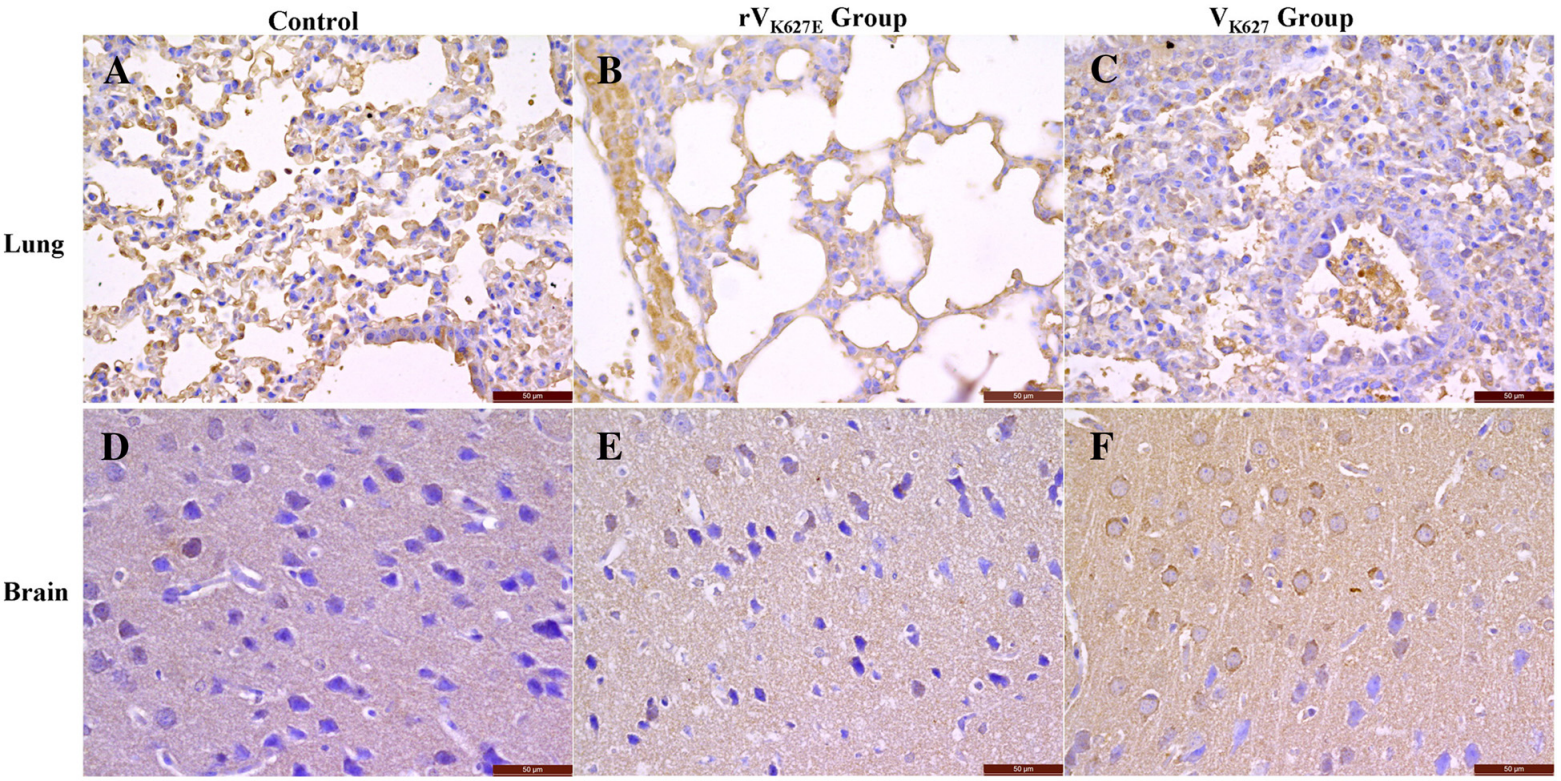
**Distribution of NLRP3 in the lung and brain of infected BALB/c mice and the control at 5 dpi.** Scale bar = 50 μm. **A**, **B** and **C**: Lung of control, rV_K627E_ and V_K627_ group, respectively; **D**, **E** and **F**: Brain of control, rV_K627E_ and V_K627_ group, respectively.

Immunohistochemical detection was performed to show the cell and tissue specific distribution of the NLRP3, IL-1β and TNF-α in the lung and brain. For the control, the positive cells and distribution of NLRP3 in lung and brain were consistent with our previous study [28] (Figure 2A). The infiltrated inflammatory cells and exudation were positive in the lung of infected groups (Figure 2B and C). In the cerebral cortex of brain, neurons showed medium positive staining in the control and rV_K627E_ group, while stronger positive staining was detected in V_K627_ group (Figure 2D, E and F).

**Figure 3:**
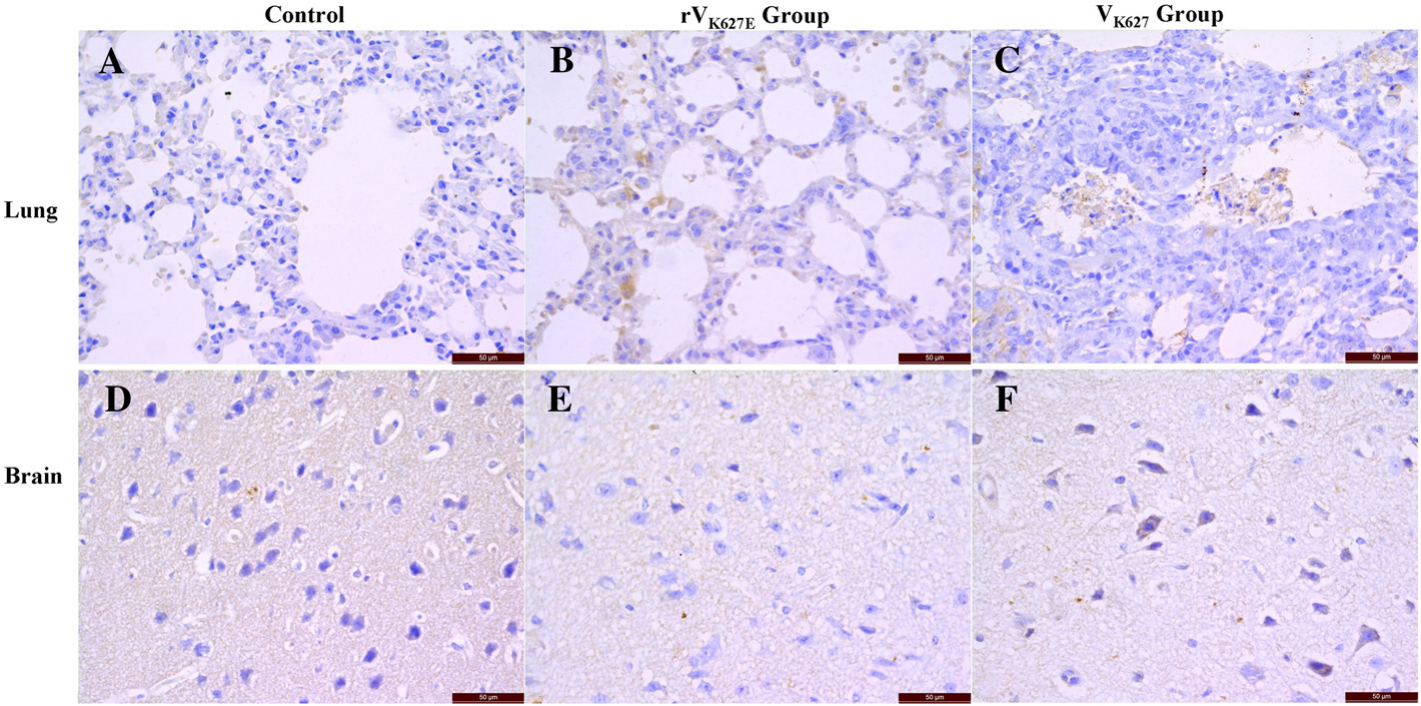
**Distribution of IL-1β in the lung and brain of infected BALB/c mice and the control at 5 dpi.** Scale bar = 50 μm. **A**, **B** and **C**: Lung of control, rV_K627E_ and V_K627_ group, respectively; **D**, **E** and **F**: Brain of control, rV_K627E_ and V_K627_ group, respectively.

IL-1β in the lung of the control showed some alveolar epithelial cells of the pulmonary alveoli and bronchiole were very weak positive (Figure 3A), while mild to strong positive of the same cells in rV_K627E_ and V_K627_ groups (Figure 3B and C). In the cerebral cortex of the brain, neurons showed negative staining and only punctiform positive in matrix in the control and rV_K627E_ group, (Figure 3D and E), while strong positive staining in neuron cells with punctiform positive in matrix in V_K627_ infection group (Figure 3F).

**Figure 4:**
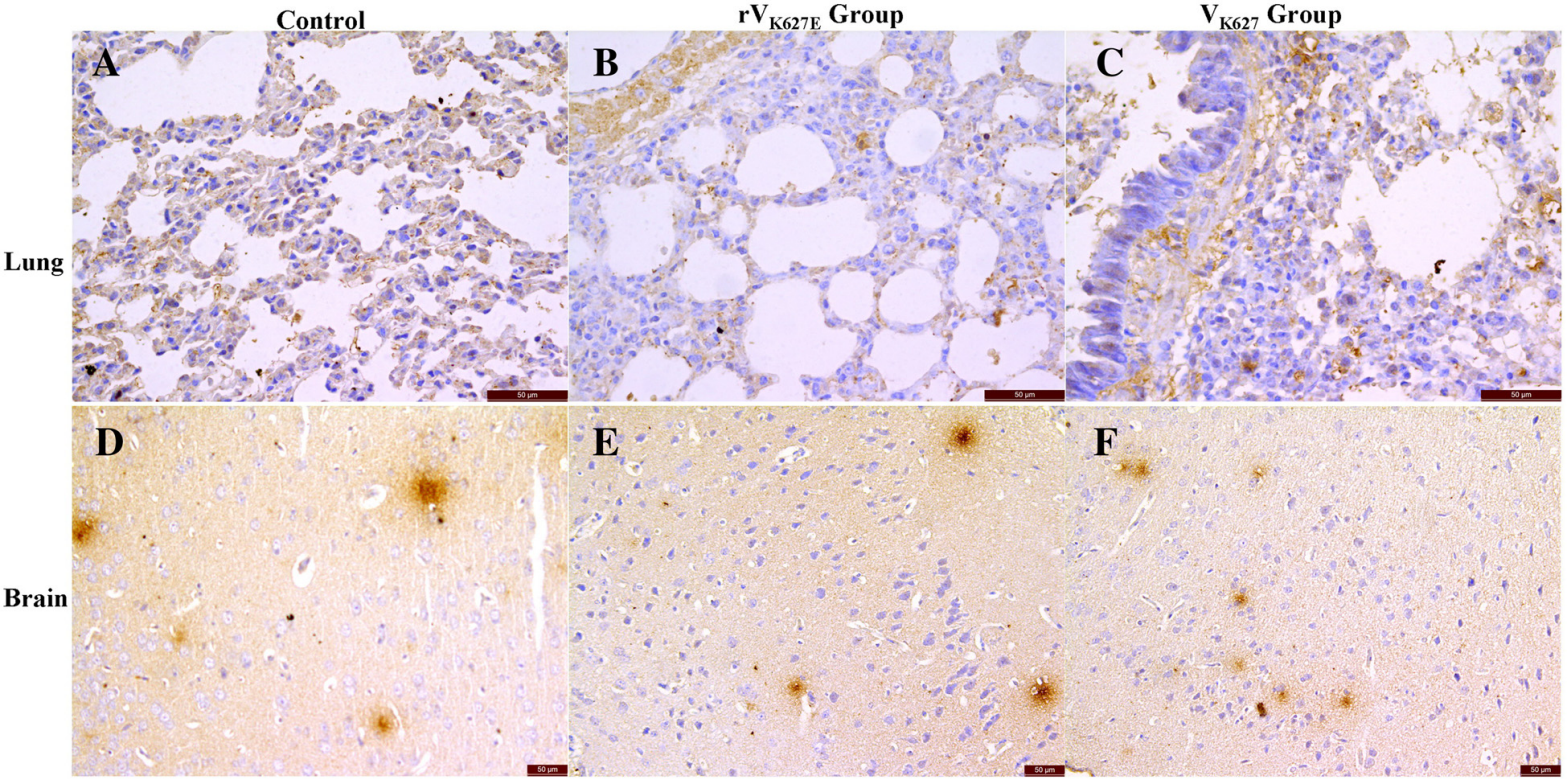
**Distribution of TNF-α in the lung and brain of infected BALB/c mice and the control at 5 dpi.** Scale bar = 50 μm. **A**, **B** and **C**: Lung of control, rV_K627E_ and V_K627_ group, respectively; **D**, **E** and **F**: Brain of control, rV_K627E_ and V_K627_ group, respectively.

In the lung, the TNF-α positive cells were mainly the epithelial cells of the pulmonary alveoli in mice of the control (Figure 4A), while stronger positives in the epithelial cells of the pulmonary alveoli as well as the epithelial cells and the submucosa of bronchi and bronchioles in both V_K627_ and rV_K627E_ infected mice (Figure 4B and C). Notably, the shed bronchial epithelial cells, intraluminal secretions and infiltration cells in the alveolar wall and around the bronchi and bronchioles are strong positives in the lung of infected groups (Figure 4B and C). In the brain, TNF-α were focal expression of matrix and there was no significant difference between infected groups and the control (Figure 4D, E and F).

## Discussion

Mice are routine laboratory animals to characterize host responses during influenza virus infection [29] and different kinds of mice exhibit different susceptibilities and immune responses during infection [27]. Otte *et al.* reported that BALB/c mice were more sensitive to H5N1 [30] which recombined from H9N2 [17,18] than C57BL/6 mice. Our previous researches proved that H9N2 avian influenza virus strains with only single-amino-acid substitution in PB2 residue 627 had different pathogenicity in BALB/c mice [21,22]. The relationship between this pathogenesis change and inflammation active factor NLRP3 is still obscure. As the lung and brain showed significant differences in these two H9N2 avian influenza virus strains infection, we chosen to detect the expression patterns of NLRP3 and its related cytokines of IL-1β and TNF-α.

The data of expression level of NLRP3, IL-1β and TNF-α in lung showed that they were changed by H9N2 avian influenza virus infection. In infected groups, the expression level of NLRP3, IL-1β and TNF-α increased rapidly at 3 dpi and V_K627_ infection were higher than that of rV_K627E_. These data implied that NLRP3 inflammasome and its signal pathway in lungs had been activated by H9N2 AIV infection and the site 627 contributed to these changes. The histopathological changes of the lung also proved this. In the brain, the expression level of NLRP3 and IL-1β in V_K627_ group increased rapidly while rV_K627E_ infection not, this suggested that V_K627_ infection can activate the inflammatory processes of brain quickly while rV_K627E_ infection can not. Meanwhile, the anti-virus replication function cytokine TNF-α of rV_K627E_ groups are significant higher than the other two groups. Interestingly, expression level of TNF-α declined in lung while increased in brain at 6 dpi after infection. These information may explain that why the viral encephalitis only in V_K627_ infection while rV_K627E_ not.

Results of immunohistochemical detection showed that NLRP3, IL-1β and TNF-α were constitutively expressed in lungs and brains of BALB/c mice. When the H9N2 avian influenza virus infection occurred in BALB/c mice, there were changes in quantity. The exudates cells and liquid in lung showed positive indicate that NLRP3, IL-1β and TNF-α exist in this inflammation infiltration. To our knowledge, there are very few papers which give the tissue distribution of NLRP3, IL-1β and TNF-α for normal and influenza virus infected BALB/c mice. The cell and tissue-specific distribution of NLRP3, IL-1β and TNF-α in the BALB/c mice will help to elucidate the role of victim tissues in AIV infection.

## Conclusion

In conclusion, our data showing the expression patterns of NLRP3, IL-1β and TNF-α in H9N2 AIV infected mice further support that NLRP3 inflammasome protein play a crucial role in the physiological and pathological processes of influenza infection. Both V_K627_ and rV_K627E_ infection can active the expression of NLRP3, IL-1β and TNF-α in the lung and brain of BALB/c mice at different levels and this also improved that site 627 in PB2 takes part in the virulence of H9N2. This basic data will enhance our understanding of the inflammatory process during influenza and develop the new strategies to anti-influenza based on the NLRP3.

## Materials and methods

### Viruses and BALB/c mice infection by H9N2 influenza virus

H9N2 AIV viruses used in study were A/chicken/Guangdong/V/2008 (V_K627_) and a site-mutation virus (rV_K627E_) which was rescued by eight-plasmid reverse genetic system [21,22]. Thirty-six SPF female BALB/c mice (18.0-20.0 g, 6 weeks) from Guangdong Experimental Animal Centre, Guangzhou, China were used in this research and randomly divided into two infection and control groups. The procedures and steps of management and challenge test by this two H9N2 AIVs strains are similar to our previous reports [21,22,31]. The animal experiments were approved by the Institutional Animal Care and Use Committee at the South China Agricultural University (Certification Number: CNAS BL0011).

### Tissue preparation

Lungs and brains of three mice in each group were collected promptly at 1, 3, 5 and 6 dpi. Each tissue was divided into two parts, one for total RNA extraction and the other for histopathological and immunohistochemistry detection.

### RNA extraction, cDNA preparation and quantitative real-time PCR

The procedures and steps of total RNA, cDNA preparation and quantitative real-time PCR were similar to our previous reports [21,22,28,31]. Relative expression levels of NLRP3, IL-1β, and TNF-α using the 2^-△△Ct^ formula and endogenous housekeeping gene β-actin for normalizing. The primer sequences were synthesized as follows: 5′- ACCAGCCAGAGTGGAATGA -3′(NLRP3-Fw), 5′- GCGTGTAGCGACTGTTGAG -3(NLRP3- Rv), 5′- CTCGTGCTGTCGGACCCAT -3′(IL-1β-Fw), 5′- CAGGCTTGTGCTCTGCTTGTGA -3′(IL-1β- Rv), 5′-TCTCATTCCTGCTTGTGGC -3′(TNF-α-Fw), 5′- CACTTGGTGGTTTGCTACG-3′(TNF-α-Rv), 5′-CATCCGTAA AGACCTCTATGCCA-3′(β-actin-Fw) and 5′- ATGGAGCCACCGATCC ACA-3′ (β-actin-Fw). Statistical analysis of the data was performed by using t test and one way anove using GraphPad Prism 5 (GraphPad Software, La Jolla, CA). Data are expressed as the mean ± SD from three separate experiments.

### Histopathological and immunohistochemical detection

The procedures and steps of tissue section preparation were similar to our previous reports [28,31]. Serial sections were taken from the same sample, one for histopathological observation by H.E. staining and the other for immunohistochemical detection. After the endogenous peroxidase activity was quenched, antigen retrieval was performed and necessary washed, the slices then incubated with correspondence antibodies (antibody for NLRP3, abcam, Cat.No., ab4207; antibody for IL-1β, santa crus biotechnology, sc-7884; antibody for TNF-α, abcam, Cat.No., ab6671), diluted 1:200 for antibodies of NLRP3 and TNF-α and 1:150 for antibody of IL-1β, for 2 h at 37°C. After incubation with the correspondent secondary antibody for 1 h at 37°C, the slices were washed 3 times by PBS at 5 minutes intervals. Then used DAB (3, 3’-diaminobenzidine -tetrahydrochloride) kit for coloration and re-dyed with hematoxylin.
